# Extracting kinetic parameters for homogeneous [Os(bpy)_2_ClPyCOOH]^+^ mediated enzyme reactions from cyclic voltammetry and simulations

**DOI:** 10.1016/j.bioelechem.2008.08.001

**Published:** 2008-11

**Authors:** V. Flexer, M.V. Ielmini, E.J. Calvo, P.N. Bartlett

**Affiliations:** a INQUIMAE, Departamento de Quimica Inorganica, Analitica y Quimica Fisica, Facultad de Ciencias Exactas y Naturales, Universidad de Buenos Aires, C1428EHA, Argentina; b School of Chemistry, University of Southampton, Southampton, SO17 1BJ, UK

**Keywords:** Enzyme electrode, Mediator, Kinetics, Glucose oxidase, Osmium bipyridine

## Abstract

The homogeneous reaction between glucose oxidase and osmium bipyridine–pyridine carboxylic acid in the presence of glucose has been studied in detail by cyclic voltammetry and digital simulation.

Combination of the analytical equations that describe the dependence of the amperometric response on enzyme, substrate and co-substrate concentrations for the limiting cases with digital simulation of the coupled enzyme reaction diffusion problem allows us to extract kinetic parameters for the substrate–enzyme reaction: *K*_MS_ = 10.8 mM, *k*_cat_ = 254 s^− 1^ and for the redox mediator–enzyme reaction, *k* = 2.2 × 10^5^ M^− 1^ s^− 1^.

The accurate determination of the kinetic parameters at low substrate concentrations (< 7 mM) is limited by depletion of the substrate close to the electrode surface. At high substrate concentrations (> 20 mM) inactivation of the reduced form of glucose oxidase in the bulk solution must be taken into account in the analysis of the results.

## 1 Introduction

In the development of new redox mediated amperometric biosensors, a suitable redox mediator has to be chosen for a particular enzyme–substrate system. The selection is usually made following analysis of results from experiments on the homogeneous system [1,2]. The reasons for choosing the homogeneous system to derive such constants is that modelling of the steady-state behaviour of such systems is much easier than for the immobilized case and that the approach can be efficiently used to screen a range of possible mediators [3].

In many studies, for oxidase systems such as glucose oxidase the rate constants for the enzyme–substrate reaction are taken from measurements made under aerobic conditions, using data already available in the literature for the enzyme reaction using molecular oxygen as the natural redox partner and without further validation for the artificial mediator. This can be misleading, therefore it is important to have reliable methods to evaluate the kinetic constants for soluble artificial mediators.

The treatment given by Albery et al. [4] is one of the most generally used theoretical approaches to analyse experimental voltammetric data for the homogeneous system in order to extract the relevant kinetic constants for the reactions. For a full description of the model, the reader is referred to the original paper [4] or to the paper by Bartlett and Pratt [1] where the model has been reviewed, results of an experimental test presented and where some of the limitations have been discussed. Scheme I shows the homogeneous system as considered by Albery et al. [4].

The reactions occurring in solution are

(1) $$S+E_{\text{ox}\overset{k_{-1}}{\leftarrow }}^{\overset{k_{1}}{\to }}ES\overset{k_{\text{cat}}}{\to }P+E_{\text{red}}$$

(2) $$2M_{\text{ox}}+E_{\text{red}}\overset{k}{\to }2M_{\text{red}}+E_{\text{ox}}$$

with

(3) $$K_{\text{MS}}=\left(k_{-1}+k_{\text{cat}}\right)/k_{1}$$

At the electrode surface

(4) $$M_{\text{red}}\to M_{\text{ox}}+e^{-}$$

In this reaction scheme *S* represents the substrate and *P* the product. *E*_ox_ and *E*_red_ are the oxidised and reduced forms of the enzyme, and *M*_ox_ and *M*_red_ the oxidised and reduced forms of the mediator.

In deriving the model high substrate concentrations are assumed in order to keep substrate depletion at the electrode surface to a minimum. This greatly simplifies the mathematical treatment of the problem, but this approximation breaks down under certain conditions as has already been pointed out [1,5]. We return to this point later.

The diffusion coefficients for the oxidized and reduced forms of the enzyme are taken as equal, which means that the total enzyme concentration, *e*_Σ_, is assumed to be constant at all points throughout the solution. The same assumption is made for the oxidized, *M*_ox_, and reduced, *M*_red_, forms of the mediator.

When diffusion–reaction equations are written under steady-state conditions, it is not possible to derive a single analytical solution which is valid under all experimental conditions, since the differential equations have significant non-linear terms. Albery et al. [4] derived approximate solutions for the different limiting cases.

Two of these limiting cases of the five in the case diagram [1,4], are relevant to the present work and will be used to derive kinetic data from experimental results for the homogeneous system. Following the notation of the original paper [4], these are Cases I and VI.

Case I corresponds to mediator–enzyme limited kinetics where the oxidised form of the mediator *M*_ox_ generated at the electrode is consumed within the diffusion layer by reaction with the reduced form of the enzyme present at its bulk concentration in a first order reaction layer of thickness (*D*_M_/*ke*_Σ_)^1/2^ adjacent to the electrode, where *D*_M_ is the diffusion coefficient for the mediator. The resulting current is first order in mediator, half order in enzyme and independent of substrate concentration. The amperometric response is given by

(5) $$I_{1}=nFAm_{\Sigma }{\left(D_{\text{M}}ke_{\Sigma }\right)}^{1/2}$$

where *m*_Σ_ is the total concentration of mediator, *e*_Σ_ is the total enzyme concentration, *k* is the rate constant for the reaction between the enzyme and the mediator (see Scheme I), *A* is the electrode area, *F* the Faraday and *n* the number of electrons transferred. This behaviour is identical to an EC′ mechanism with *k*_EC′_ = *ke*_Σ_ and was used by several groups in the early studies (Cass [6], Green [7], Liaudet [8], Rusling [9], Frede [10]) to evaluate values of *k*. Bourdillon, Saveant and co-workers [11] pointed out that a large concentration of substrate is not a sufficient condition for the pseudo-first order EC′ approximation to hold and that the condition *km*_Σ_/*k*_cat_ << 1 is also necessary. These authors worked out a close-form expression for the enzyme catalytic plateau current in cyclic voltammetry when a one-electron mediator re-oxidizes GOx(FADH_2_) assuming no substrate depletion at the electrode surface and for a glucose concentration at least 50 times larger than the soluble mediator concentration.

Eq. (5) has been verified experimentally by Pratt and Bartlett for glucose oxidase and ferrocene monocarboxylic acid [1] and by Liaudet et al. [8] for ferrocene monosulfonate. Using explicit finite difference simulation Battaglini and Calvo [12] have shown that Lineweaver Burk plots are non-linear unlike simple Michaelis–Menten kinetics, and this is the result of the interplay of kinetics and diffusion near the electrode surface.

It should be noted that in order to derive Eq (5) the assumption of negligible substrate depletion near the electrode surface has been made, thus the differential equations have been solved with the approximation *s* ≈ *s*_∞_ (where *s* is the substrate concentration at an arbitrary distance from the electrode and *s*_∞_ is the bulk substrate concentration). This approximation may break at low substrate concentration or high enzyme activities. Previous experiments and simulations [1,5], however, have shown that substrate depletion at either low substrate concentration and/or high enzyme activity should not be ignored. Substrate depletion leads to poor fitting of the experimental data and in some early work also led to significant overestimation of *K*_MS_, since this value is extracted from the low concentration range of the calibration curves.

Case VI corresponds to enzyme–substrate limited kinetics. For Case VI

(6) $$I_{\text{VI}}=nFA{\left(\frac{2D_{\text{M}}k_{\text{cat}}e_{\Sigma }m_{\Sigma }s_{\infty }}{s_{\infty }+K_{\text{MS}}}\right)}^{1/2}$$

Note that the current is half order in mediator, half order in enzyme and, when *s*_∞_ << *K*_MS_ also half order in substrate. The enzyme–substrate kinetics can be determined from calibration plots of the catalytic current as a function of the substrate concentration. The number of electrons exchanged by the [Os(bpy)_2_ClPyCOOH]^+^ mediator is *n* = 1 and the factor of 2 in the parenthesis in Eq. (6) arises from the parabolic profile for *M*. In this case, the soluble redox mediator diffuses in a reaction layer close to the electrode where the rate-limiting step for its destruction is not the reaction of mediator and the reduced form of the enzyme, but the generation of reduced enzyme GOx(FADH_2_) from GOx(FAD) resulting in a much lower concentration of GOx(FADH_2_) than the bulk concentration so that *e*_ox_ ≈ *e*_Σ_ (where *e*_ox_ is the concentration of oxidized enzyme) and the zero-order reaction layer thickness is given by

(7) $$X_{k}={\left[\frac{D_{\text{E}}}{k_{\text{cat}}}\left(\frac{K_{\text{MS}}}{s_{\infty }}+1\right)\right]}^{1/2}$$

where *D*_E_ is the enzyme diffusion coefficient. A non-linear least squares fit of a plot of catalytic current as a function of substrate concentration over a wide range of concentrations should yield the values of *K*_MS_ and *k*_cat_, if the conditions for Case VI are met.

A further factor to consider in modelling amperometric enzyme electrodes is the inactivation of the soluble enzyme. In amperometric enzyme electrodes with HRP immobilized at the electrode, Saveant and co-workers have considered inactivation of the enzyme [13] but, to the best of our knowledge, this has not been taken into account in modelling biosensors or when determining the kinetic parameters from electrochemical data for dissolved enzyme.

Nevertheless, it is not unusual to find reports of the decay of amperometric biosensor response with time over timescales ranging from a few hours to several days. Moreover, there are several reports of the inactivation of glucose oxidase (GOx)—the most widely used redox enzyme in the biosensor field [14–21]. Miron et al. [20] have analyzed the different reasons why some authors have paid more attention to GOx inactivation than others.

In the present work we report a combined strategy to obtain the kinetic constants *k*, *k*_cat_ and *K*_MS_ that characterize the homogeneous system β-d-glucose–glucose oxidase, glucose and [Os(bpy)_2_ClPyCOOH]^+^ (the structure of the redox mediator is shown in Scheme II). We first try to obtain the kinetic constants in the classical way using Albery's approximate analytical equations for selected limiting cases in the case diagram [4]. We then discuss the limitations of this method and try to improve the data analysis by comparing the experimental voltammograms with numerical simulations in order to obtain more accurate estimates of the rate constants.

Finally we also report how inactivation of glucose oxidase affects the electrochemical results and analysis.

## 2 Experimental

A standard three-electrode electrochemical cell was employed with an operational amplifier potentiostat (TEQ-Argentina). An Ag/AgCl; 3 M KCl (0.210 V vs. NHE) reference electrode was employed and all electrode potentials herein are referred to it; the auxiliary electrode was a large area platinum gauze. All electrochemical experiments were carried out at room temperature (20 ± 2)°C. Argon was used to sparge all solutions to remove dissolved oxygen taking special care not to produce excessive foam and segregation of the enzyme at the liquid–air interface which could lead to denaturation of the enzyme. Unless otherwise specified, electrochemical measurements were carried out in 0.1 M NaH_2_PO_4_/Na_2_HPO_4_, 0.1 M NaCl buffer solutions of pH 7.

The working electrode was a home-made glassy carbon electrode (0.5 cm in diameter) encased in epoxy resin (Araldite^®^).

Two preparations of Glucose oxidase (GOx E.C. 1.1.3.4) from *Aspergillus niger* were used. The majority of experiments were carried out with the enzyme from Fluka (molar mass, 186,000 Da). The second enzyme sample was a generous gift from MediSense^®^, UK (molar mass, 156,000 Da). Both were used without further purification.

The molecular weight of the enzymes was determined by gel electrophoresis. SDS/PAGE was performed in 10% acrylamide gel, according to the method of Laemmli [22]. Protein samples were denatured by a 5 min incubation with SDS reducing buffer (62.5 mM Tris–HCl; 10% glycerol; 2% SDS; 0.1 M DTT, 0.01% bromophenol blue, pH 6.8) at 95 °C. Proteins were stained with Coomassie^®^ brilliant blue G250.

The electrophoresis gel was calibrated using a protein standard mixture (Sigma High Molecular Weight standard) containing myosin (205 kDa), β-galactosidase (116 kDa), phosphorylase b (97 kDa), bovine albumin, (66 kDa), ovalbumin (45 kDa) and carbonic anhydrase (30 kDa).

β-d-glucose, NaH_2_PO_4_, Na_2_HPO_4_, KH_2_PO_4_, K_2_HPO_4_, KNO_3_ and NaCl (Merck) and L-glucose (Fluka) were used as received. All glucose solutions were stored for a minimum of 24 h at 4 °C to allow equilibration of the anomers [1].

The complex [Os(bpy)_2_ClPyCOOH]^+^ (where PyCOOH is pyridine-carboxylate) was prepared as previously reported [23].

The simulation program was written in Borland^®^ Turbo Pascal v 5.5 [24] and is based on the explicit finite difference method [25,26] assuming steady state for the enzyme kinetics. Mass transport of the soluble enzyme was neglected since its diffusion coefficient (*D*_E_ = 5 × 10^− 7^ cm^2^ s^− 1^) is significantly less than that of the soluble mediator and substrate. The program also takes into account the equilibrium of the two glucose anomers, and allows substrate depletion to be taken into consideration [24].

## 3 Results and discussion

### 3.1 Approximate limiting cases

**Fig. 1:**
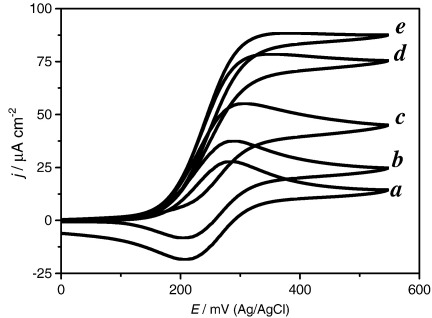
Cyclic voltammograms recorded at different d-glucose concentrations and constant *m*_Σ_ = 1.02 mM and *e*_Σ_ = 1.6 μM in NaH_2_PO_4_/Na_2_HPO_4_ 0.1 M + 0.1 M NaCl buffer solution. Glucose concentration (mM): a) 0.0; b) 1.6; c) 3.2; d) 9.9; e) 22.7. The reference electrode is Ag/AgCl in 3 M KCl, scan rate 5 mV s^− 1^.

Fig. 1 depicts a typical set of cyclic voltammograms recorded at 5 mV s^− 1^ in a solution containing 1.0 mM [Os(bpy)_2_ClPyCOOH]^+^ and 1.6 µM GOx (Fluka) in 0.1 M NaH_2_PO_4_/Na_2_HPO_4_, 0.1 M NaCl buffer solution of pH 7 in the absence (a) and in the presence of increasing concentrations of d-glucose, the enzyme–substrate, (b–e). Fig. 1a shows a typical reversible cyclic voltammogram and the diffusion limited peak current for the oxidation of the Os(II) complex is described by the Randles Sevčik equation [27] with an *E*′ of 243 mV and *D*_M_ of 2.7 × 10^− 6^ cm^2^ s^− 1^. In the presence of glucose the shape of the voltammetric curve evolves towards a catalytic wave but significant hysteresis between the forward and backward potential sweeps is always observed (Fig. 1b–e).

**Fig. 2:**
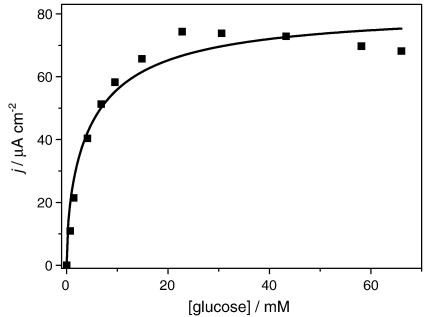
Plot of current as a function of glucose concentration in case VI. *m*_Σ_ = 1.02 mM, *e*_Σ_ = 1.6 μM in NaH_2_PO_4_/Na_2_HPO_4_ 0.1 M + 0.1 M NaCl buffer solution. The line shows the best fit to Eq. (6) of the experimental data. All experiments carried out in the same solution with successive additions of more d-glucose.

Our first approach to analyse the data is to extract the enzyme–substrate kinetic constants, *K*_MS_ and *k*_cat_ from a classic calibration plot of the catalytic current against glucose concentration, Fig. 2. The experimental catalytic currents plotted in Fig. 2 were taken from the plateau currents in the cyclic voltammograms at 0.50 V for measurements at 5 mV s^− 1^.

Note the maximum current and the current decay at high concentrations, we return to this later.

The value of the apparent Michaelis constant *K*_MS_ is usually calculated in one of two ways. i) *K*_MS_ is taken as the substrate concentration at which the current is half of the limiting current [28]. This assumes a first order dependence of the current on substrate concentration, however the current is in fact half order in substrate for case VI (see Eq. (6)). ii) For *s*_∞_ << *K*_MS_, Eq. (6) yields a linear dependence on the square root of the substrate concentration

(8) $$I=nFA{\left(2D_{\text{M}}\frac{k_{\text{cat}}}{K_{\text{MS}}}e_{\Sigma }m_{\Sigma }s_{\infty }\right)}^{1/2}$$

**Fig. 3:**
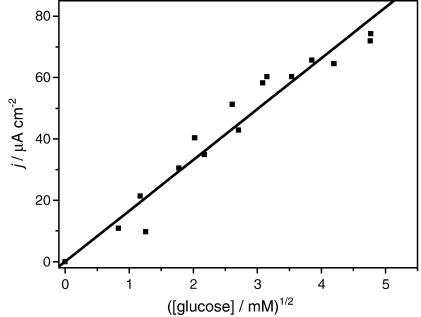
Plot of current as a function of the square root of substrate concentration. *m*_Σ_ = 1.02 mM, *e*_Σ_ = 1.6 μM in NaH_2_PO_4_/Na_2_HPO_4_ 0.1 M + 0.1 M NaCl buffer solution. The line is the best linear fit for the data points that adjust better to the linear regime.

Thus a plot of *I* against *s*_∞_^1/2^ should give a straight line passing through the origin at low *s*_∞_, as shown in Fig. 3. From the gradient of this line we can obtain a value for the ratio *k*_cat_/*K*_MS_. From the slope of Fig. 3 we obtained *k*_cat_/*K*_MS_ = 3.9 × 10^3^ M^− 1^ s^− 1^ however this is not a reliable value due to inactivation of the enzyme as discussed below.

The value of the rate constant *k*_cat_ is normally calculated from the current in the saturated region, *I*_MAX_, i.e. when *s*_∞_ >> *K*_MS_

(9) $$I_{\text{MAX}}=nFA{\left(2D_{\text{M}}k_{\text{cat}}e_{\Sigma }m_{\Sigma }\right)}^{1/2}$$

In the present study, however, it has been found that the catalytic current passes through a maximum with increasing substrate concentration and then declines rather than reaching a constant limiting value This precludes the extraction of reliable enzyme kinetic data in the usual way. For the same reason non-linear fitting of the experimental data in Fig. 2 to Eq. (6) is very poor (solid line), since Eq. (6) predicts a constant value at high glucose concentration. Low values of *K*_MS_ = 13 ± 5 mM and *k*_cat_ = 60 ± 20 s^− 1^ are obtained from the fitting.

Also, notice in Fig. 2 that for very low glucose concentrations, appreciable depletion might lead to an error in the values estimated as will be shown by the digital simulation results below.

### 3.2 Catalytic current decay

It is clear from Fig. 2 that the catalytic current as a function of substrate concentration passes through a maximum. To determine the cause of this we need to consider the experimental conditions in detail.

First, contamination of the electrode surface was ruled out by polishing the electrode while keeping the same electrolyte solution containing enzyme, substrate and co-substrate. The concentration of the stable Os complex was also monitored by UV–visible spectroscopy at regular times after additions of d-glucose. As shown in Fig. S-1 of the Supporting Information, no change in the maximum peak absorbance for the Os complex was found.

Since the decay in catalytic current is more evident at high substrate concentrations, it could result from a change in the local pH at the electrode surface [16] since the enzyme kinetics are known to be pH dependent [29,30]. There is an important difference between the aerobic and the anaerobic re-oxidation of GOx. In the first case, during glucose oxidation the two protons produced in the reaction are taken by the enzyme prosthetic group to yield GOx(FADH_2_) and during the aerobic re-oxidation of GOx(FADH_2_), molecular oxygen consumes these two protons to yield H_2_O_2_. In anaerobic re-oxidations of GOx(FADH_2_) by one-electron outer sphere redox couples, such as the Os complex, the redox mediator does not consume the protons and hence they remain as a by-product in solution decreasing the pH at the electrode surface [16]. However we can also rule this out as a possible explanation since when the calibration experiment was repeated in more concentrated 0.5 M buffer solution there was no appreciable difference in the result (see Fig. S-2 in Supporting Information).

A further possibility is that the diffusion coefficient of the redox mediator is decreased due to an effect of the viscosity of the solutions at high glucose concentration. This can be ruled out since a plot of peak current vs. square root of the scan rate [27] for the soluble Os complex in the absence of the enzyme in pure buffer and in buffer with 100 mM glucose shows only a slight variation of less than 2% (see Fig. S-3 in Supporting Information).

**Fig. 4:**
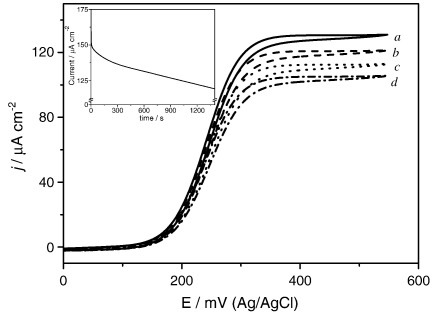
Cyclic voltammograms for *m*_Σ_ = 1.05 mM, *e*_Σ_ = 2.4 μM, *s*_∞_ = 46 mM in 0.1 M NaH_2_PO_4_/Na_2_HPO_4_ + 0.1 M NaCl buffer solution. a.) first CV at time 0; b.) voltammogram after 5 min; c.) CV after 10 min; d.) CV after 25 min. The inset shows a chronoamperometry transient at 0.50 V. (*m*_Σ_ = 1.22 mM, *e*_Σ_ = 2.6 μM, *s*_∞_ = 117 mM, same buffer) showing the current decay in time without addition of any further reactants. The reference electrode is Ag/AgCl in 3 M KCl, scan rate 5 mV s^− 1^.

Next we investigated enzyme inactivation as a possible cause of the catalytic current decay. A clear drop in catalytic current with time can be observed in Fig. 4 for successive cyclic voltammetry experiments, taken every few minutes, without further additions of any reactants. Surprisingly, the rate of inactivation is rather fast. The inset in Fig. 4 is a chronoamperometry experiment over 25 min at a potential of 0.50 V. Note that this decay in current with time cannot be accounted for by consumption of the glucose by the electrode reaction; at the highest rate the glucose concentration in the cell will decrease for 50 mM to only 49.92 mM in 15 min.

**Fig. 5:**
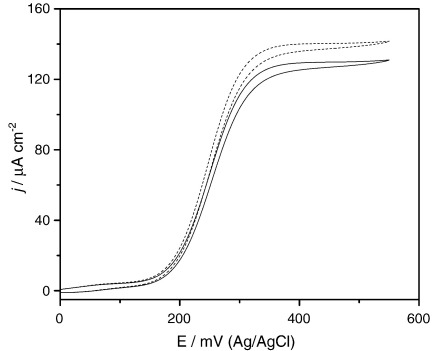
Cyclic voltammograms for *m*_Σ_ = 1.02 mM, *e*_Σ_ = 1.6 μM in NaH_2_PO_4_/Na_2_HPO_4_ 0.1 M + 0.1 M NaCl buffer solution. Full line is in the presence of 100 mM d-glucose. Dotted line is in the presence of 50 mM d-glucose solution and was measured 10 min after the first voltammogram as described in the text. The reference electrode is Ag/AgCl in 3 M KCl, scan rate 5 mV s^− 1^.

Finally, the sequence of measurement steps was varied as follows: the original buffer solution containing the Os complex and GOx was divided into two aliquots. Glucose was added to the first aliquot up to a concentration of a 100 mM and a voltammogram was recorded on a clean electrode. Then, in order to test the enzyme that had not been in contact with the substrate, the second aliquot of the original solution was added, keeping buffer, Os complex and GOx concentrations unchanged. A new voltammogram on the same electrode showed a clear increase in the catalytic current for the more dilute d-glucose with fresh enzyme solution as shown in Fig. 5. Thus, we can conclude that the inactivation of the enzyme only takes place in the presence of its substrate glucose.

Our experiments have shown that the effect is not due to electrode fouling, decomposition of the Os complex or changes in local pH. Therefore we conclude that the enzyme suffers some form of inactivation.

When the mediator was changed to ferrocene methanol a decay (though less pronounced) in the catalytic current at high glucose concentration was also seen (see Fig. S-4 in Supporting Information).

There is some evidence in the literature for the inhibitory effects of Na^+^ on GOx [31], albeit on the time scale of hours, but inhibition has not been observed in the presence of K^+^. In our hands a glucose calibration experiment under the same conditions as Fig. 2 but in 0.1 M KH_2_PO_4_/K_2_HPO_4_ + 0.1 M KNO_3_ buffer solution showed the same effect.

In a recent detailed paper, Miron et al. [20] suggested that the reason why GOx inactivation in solution is sometimes neglected could be due to structural differences between enzymes of different origin. We have repeated the calibration experiment with GOx from *Aspergillus niger* from a different source with a different degree of glycosilation. As shown in the (Supporting Information Fig. S-5), the decay in catalytic current at high d-glucose concentration can still be seen but is much less significant than in the case of the enzyme from Fluka.

The decay in catalytic current with time (inset in Fig. 4) supports the idea of an auto-inactivation of the soluble enzyme since it has been observed even if the catalysis is interrupted for some time.

Substrate inhibition has been described for the free enzyme from *Aspergillus niger* in the presence of excess substrate (Miron et al. [20] and references therein). The decay in the current observed in our work seems to be associated with the presence of the reduced form of the enzyme, and not necessarily with the number of catalytic cycles the enzyme undergoes. In the experiments described in this paper, GOx is in solution in its oxidised form and the Os complex in its reduced form. Upon addition of d-glucose the enzyme is reduced and it remains in the reduced state unless it approaches the electrode surface where it can be oxidised by two mediator molecules regenerating the oxidised enzyme. Therefore, only the small fraction of enzyme molecules (of the order of 1 in 10^12^ to 10^14^) in the vicinity of the electrode will be re-oxidized and undergo several catalytic cycles while most of GOx remains in the reduced form throughout the experiment. From the choronoamperometry experiment we calculated a first order inactivation rate constant of 2.9 × 10^− 4^ s^− 1^.

**Fig. 6:**
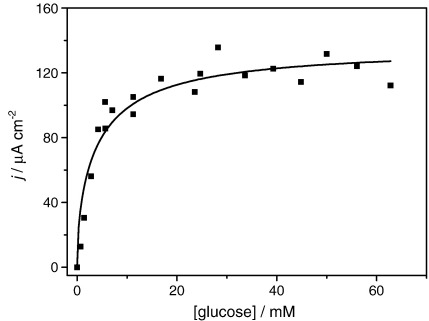
Plot of current as a function of glucose concentration in case VI. *m*_Σ_ = 1.02 mM, *e*_Σ_ = 1.6 μM in NaH_2_PO_4_/Na_2_HPO_4_ 0.1 M + 0.1 M NaCl buffer solution. Full line shows the best fit to Eq. (6) of the experimental data. Every data point corresponds to a new buffer/mediator/GOx/d-glucose solution that was discarded after taking the experimental CV.

To avoid the time dependent effects of the enzyme inactivation processes, we redesigned the experimental protocol so that all the glucose concentration data points were measured after the same time. We freshly prepared new enzyme, Os complex and buffer solution, added glucose to the desired concentration, then measured the voltammogram and discarded the solution. In this way, the enzyme was in contact with the substrate for the shortest time possible in all cases. Fig. 6 shows the new calibration plot obtained in this way. There is greater dispersion of the data points in Fig. 6 than in Fig. 2, due to slight variations in concentration because new solutions were prepared for every data point. However, it is noticeable that the data in Fig. 6 no longer shows evidence for a fall in current at high glucose concentration and is in much better agreement with the proposed model given in Eq. (6) as can be seen from Fig. 6. Notice also that larger current densities than in Fig. 2 are observed for the same glucose concentrations.

This new experimental design was also used for the experiments used to study Case I.

**Fig. 7:**
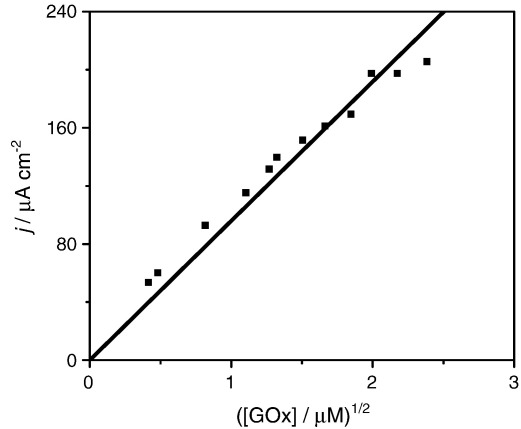
Plot of catalytic current as a function of the square root of enzyme concentration at d-glucose saturation and best linear fit of the experimental data points. *m*_Σ_ = 1.02 mM, *s*_*∞*_ = 45 mM in NaH_2_PO_4_/Na_2_HPO_4_ 0.1 M + 0.1 M NaCl buffer solution.

The enzyme–mediator re-oxidation constant, *k*, was obtained from the slope of a plot of *I*_cat_ as a function of the square root of the enzyme concentration, *e*_Σ_^1/2^, at constant mediator concentration (1.0 mM) and under substrate saturation conditions, as shown in Fig. 7. In the calculation *D*_M_ = 2.7 × 10^− 6^ cm^2^ s^− 1^ was used. This approach is usually valid for low and moderate enzyme concentrations, since at high enzyme concentration, the plot departs from linearity due to significant substrate concentration depletion. The best fit of the experimental data in Fig. 7 to Eq. (5) yields a value for *k* of 2.1 × 10^5^ M^− 1^ s^− 1^.

### 3.3 Comparison of simulated and experimental cyclic voltammograms

The approximate analytical solutions, Eqs. (5) and (6), work well as long as the system is well away from the case boundaries where pairs of limiting cases meet [1,4] and provided that substrate depletion is not significant. We can avoid these limitations if we use numerical methods to simulate the voltammetry. The program takes into account equilibrium between the two glucose anomers, and allows for substrate depletion [24].

In this section we compare the experimental voltammetric results with simulations. In all more than 100 experimental voltammograms were compared to the simulated curves by iteratively adjusting the three kinetic parameters: *K*_MS_, *k*_cat_ and *k*. Initial values of *K*_MS_, *k*_cat_ and *k* from the approximate limiting cases were used. Literature values for the diffusion coefficients of d-glucose and the ratio of α and β anomers in an equilibrated solution were used in the simulation. For the diffusion coefficient of the redox mediator, the concentrations of d-glucose, redox mediator and total enzyme, and the redox mediator potential *E*_o_, experimental data was used.

Since there are three parameters to be adjusted, sometimes good agreement can be obtained with more than one set of parameters. Therefore we tried to constrain the kinetic constants, first within an interval centred in the estimated values and second, by selecting the conditions of lower error as best estimates.

The experimental voltammograms covered a wide range of enzyme (from 0.1 to 5.7 μM), mediator (from 0.9 to 3.2 mM), and substrate (from 0.7 to 100.0 mM), concentrations. In all cases it was possible to fit the experimental and simulated curves with the three variable kinetic parameters *K*_MS_, *k*_cat_ and *k* kept within the values given in Table 1; i.e. the same kinetic constants (± 10%) were used for over 100 voltammograms measured for very different concentrations, and corresponding to different kinetic limiting cases.

**Fig. 8:**
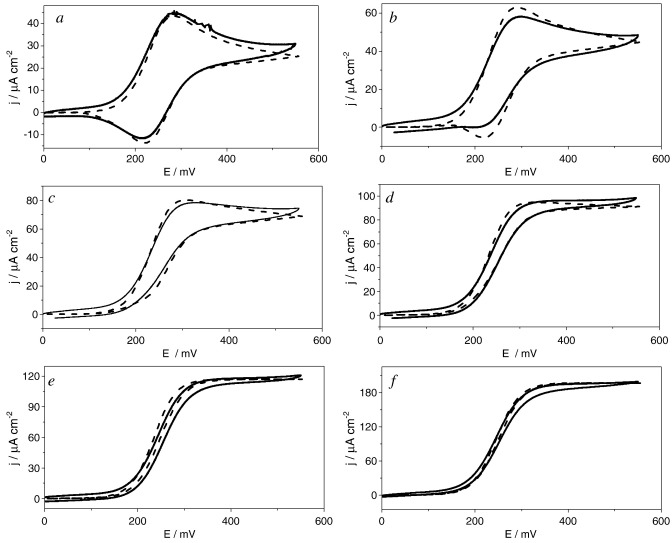
Comparison of experimental (full line) and numerical simulations (dotted line) of cyclic voltammograms for *m*_Σ_ = 1.0 mM; in 0.1 M NaH_2_PO_4_/Na_2_HPO_4_ + 0.1 M NaCl buffer solution of pH 7.0, scan rate 5 mV s^− 1^. From a to e same enzyme concentration *e*_Σ_ = 1.6 μM and different d-glucose concentrations (mM): a) 0.7; b) 1.4; c) 2.8; d) 7.0; e) 33.6; f) *e*_Σ_ = 4.72 μM and *s*_∞_ = 50.0 mM.

Fig. 8 shows a comparison of typical experimental cyclic voltammetric data and simulation results. Panels a to e in Fig. 8 correspond to the same mediator and enzyme concentration and increasing substrate concentration (see figure caption). Panel f corresponds to a different enzyme concentration and glucose saturation.

It is important to emphasise that the six simulated voltammograms were obtained using the same set of values of *K*_MS_, *k*_cat_ and *k* (± 10%).

It is apparent from Fig. 8 that the shape of the cyclic voltammograms changes significantly with glucose concentration. At low glucose concentration a peak shaped voltammogram is observed with pronounced hysteresis between the anodic and cathodic sweep directions. At higher glucose concentrations, the expected catalytic wave is observed with minimum hysteresis.

Very good agreement between the experimental and simulated curves is observed over the whole potential interval. Note that the simulation is valid in all cases even in regions of the case diagram close to the boundaries between cases where the limiting analytical equations fail to properly describe the experimental data.

**Fig. 9:**
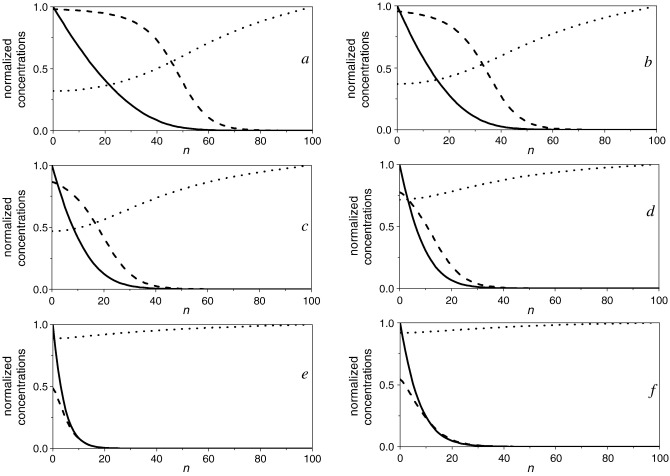
Simulated dimensionless concentration profiles at the end of the forward sweep of the cyclic voltammogram for the oxidised mediator (solid line), the oxidised enzyme (dashed line); and the enzyme–substrate glucose (dotted line) as a function of distance to the electrode (box number). Same conditions as the corresponding panel in Fig. 8.

For the conditions in Fig. 8, the concentration profiles of oxidised enzyme, Os(III) mediator and substrate in the direction normal to the electrode surface have been calculated at the end of the forward sweep. These are shown in Fig. 9.

### 3.4 Effect of substrate depletion

As already reported elsewhere [1,5,32,33], substrate depletion at the electrode surface has been observed in cyclic voltammetry at the lowest substrate concentrations.

A clear indication of substrate depletion is provided by the shape of the voltammograms at the lowest substrate concentrations. At the beginning of the potential sweep the substrate is at its bulk concentration, *s*_∞_, at the electrode surface and all the redox mediator is in the reduced form. As the potential is made more positive the concentration of Os(III) at the electrode surface increases in a Nernstian fashion. The rate of the homogeneous reduced enzyme–oxidised mediator reaction is relatively high, decreasing the concentration of glucose in the solution adjacent to the electrode resulting in a peak current above the limiting catalytic current. Above the peak potential, the concentration of oxidised mediator is fairly constant at the electrode surface while the glucose concentration continues to drop, and in the backward sweep is depleted at the electrode. The observed hysteresis is due to a lower current during the backward sweep as compared to the current on the forward sweep at the same potential until the equilibrium potential of the Os(III)/Os(II) redox couple. However, negative of this equilibrium potential no cathodic wave is observed since the Os(III) species at the surface are depleted by reaction with the reduced enzyme. Increasing the redox mediator concentration even further (above 1 mM) a cathodic peak develops in the backward sweep and reaches the same peak height of the anodic peak at zero glucose concentration as expected.

The concentration profiles show significant substrate depletion at the electrode at low concentration consistent with the observed hysteresis in the catalytic current (Fig. 9, panels a–d); and almost no depletion at the highest glucose concentration.

Notice also that the enzyme is in its oxidised form only in a narrow region close to the electrode surface while it remains in its reduced form outside the enzyme–mediator reaction layer. At higher substrate concentration this reaction layer is compressed towards the electrode surface and the concentration of the oxidized enzyme at the surface is progressively lower. As we increase the glucose concentration above *K*_MS_ the enzyme–substrate reaction becomes faster and we move towards the border with case I.

### 3.5 Comparison of kinetic data

Table 1 shows a comparison of the kinetic data, *k*_cat_, *K*_MS_, and *k*, for homogeneous enzyme kinetics mediated by the soluble osmium bipyridyl–pyridine complex derived using the different approaches we have discussed. The results obtained from the simulations and from the approximate analytical expressions are in very good agreement. However the values of *k*_cat_/*K*_MS_ are considerably larger that the value obtained from the simple analysis in Fig. 3 and this is because of the effect of inactivation of the enzyme in the earlier experiments.

Selected values for the three kinetic constants for β-d-glucose/GOx and different redox mediators are shown in Table 2. Whilst it is expected that the values for *k* change for the different redox mediators one would expect that the values for *k*_cat_ and *K*_MS_ which describe the reaction of the enzyme with glucose would be the same, according to the generally accepted ping-pong mechanism for the reaction. [29,30,34–38]. However, the values in Table 2 for both *k*_cat_ and *K*_MS_ show considerable variation while the ratio *k*_cat_/*K*_MS_ agree fairly well, particularly the data for the ferrocene mediators.

The classical work on GOx mechanism and kinetics was done at pH 5.6, which is the optimal pH for reaction with the natural mediator, O_2_ [30]. Bright et al. have shown that all kinetic constants vary significantly with the pH [29,36]. Work carried out in the biosensor field, is usually conducted at pH 7. In an early work, Bright et al. [29] reported *k*_cat_ = 900 s^− 1^ and *K*_MS_ = 68 mM at pH 7. However, many authors have compared their results obtained at pH 7 with *K*_MS_ values around 20–30 mM, which is the value reported for *K*_MS_ at pH = 5.6. The value of *K*_MS_ = 68 mM at pH 7 was obtained from spectrophotometric measurements with stopped flow and steady-state techniques for the overall reaction, unlike the results for the artificial redox mediators shown in Table 2, which were obtained with electrochemical techniques.

Yokoyama et al. [32] reported similar values for *K*_MS_ (within experimental error) but different values for *k*_cat_ for three different ferrocene mediators and suggested that the lowest *k*_cat_ value, for (ferrocenemethyl)trimethylammonium could be due to the positive charge on the mediator molecule, which can electrostatically associate with the negatively charged enzyme. This could also be the case for our redox mediator, which also carries a positive charge.

We find three possible experimental effects that could lead to apparent differences in *K*_MS_ and *k*_cat_: i) effects of substrate depletion, ii) low solubility of the redox mediator, iii) enzyme inactivation.

Substrate depletion leads to lower currents than expected at the lower substrate concentrations and hence, would result in apparent high values for *K*_MS_. The values of *k*_cat_, however, should be independent of glucose depletion since they are obtained from the limiting current at saturating glucose.

The ferrocene compounds in Table 2 are only moderately soluble in aqueous solution. In order to be well away from the border between cases I and VI, the condition *m*_Σ_ > *k*_E_/*k*, where *k*_E_ = *k*_cat_*s*_∞_/(*K*_MS_ + *s*_∞_), should be fulfilled [1,39]. If this is not the case, lower apparent values of both *k*_cat_ and *K*_MS_ would be obtained, since the maximum current at glucose saturation will be lower than the expected current for experiments performed well inside case VI. In the case of [Os(bpy)_2_ClPyCOOH]^+^, as well as for most of the data reported in Table 2, experiments would need to have been done at concentrations well beyond the solubility of the redox mediator to be well into case VI. Due to solubility limitations, however, the data was measured near the boundary with case I, and hence the kinetics are not fully enzyme–substrate limited. At the higher glucose concentrations, the enzyme–mediator reaction is not fast enough and a lower plateau current is reached. In our hands, a calibration curve in a [Os(bpy)_2_ClPyCOOH]^+^ saturated solution (solubility 3.2 mM vs. 1.0 mM as most of the experiments shown in this work) yielded a slightly higher value for *k*_cat_ (300 ± 20 vs. 233 ± 22 s^− 1^).

Enzyme inactivation leads to low apparent values for both *k*_cat_ and *K*_MS_ since the true plateau current would be never reached. Our value for *K*_MS_ is rather low when compared to other values shown in Table 2, this might be related to rather high inactivation rate observed for our enzyme.

Finally, we cannot rule out the effects of variation in the enzyme kinetics for glucose oxidase from different suppliers arising, for example, from differences in glycosylation of the enzyme [1,5,20,37,39].

## 4 Conclusions

The reaction of glucose oxidase and osmium bipyridine–pyridine carboxylic acid, [Os(bpy)_2_ClPyCOOH]^+^ (where py = pyridine), has been studied in homogeneous solution using cyclic voltammetry and digital simulation.

The limiting analytical solutions in the case diagram [4] and the simulated cyclic voltammograms agree with experimental results for the GOx/glucose/[Os(bpy)_2_ClPyCOOH]^+^ system. We have shown that the combination of simulation and experiment is a powerful strategy to extract kinetic data and to validate calculations with limiting case equations. More than 100 separate experimental cyclic voltammograms were compared to the simulation results by iteratively adjusting the three kinetic parameters for the substrate–enzyme reaction: *K*_MS_ = 10.8 mM, *k*_cat_ = 254 s^− 1^ and for the redox mediator–enzyme reaction, *k* = 2.2 × 10^5^ M^− 1^ s^− 1^. It is important to emphasise that a sufficiently large set of experimental data covering the full range of enzyme, mediator and substrate concentrations should be used in the analysis.

During these experiments, inactivation of the enzyme in concentrated glucose solutions (*k*_*i*_ = 2.9 × 10^− 4^ s^− 1^) and significant substrate depletion at low glucose concentrations has been found. The extraction of kinetic parameters is limited at very low substrate concentrations by depletion of the substrate close to the electrode surface, while at high substrate concentrations the inactivation of the glucose oxidase in its reduced form outside the oxidised enzyme–substrate reaction zone, together with the limited solubility of the redox mediator limits the analysis.

It seems that the ratio *k*_cat_/*K*_MS_ is less influenced by the parameter extraction process than the individual values of *k*_cat_ and *K*_MS_ separately.

The combined analysis described in this work is generally applicable to a wide range of homogeneous mediated enzyme systems.

## Figures and Tables

**Scheme I:**
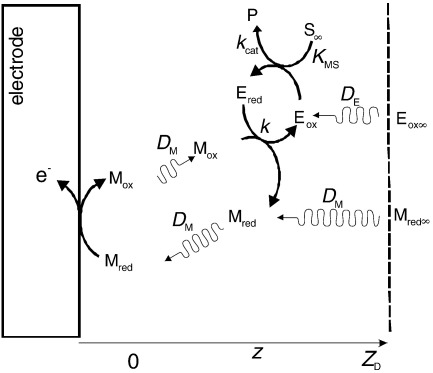
The homogeneous system as considered in references [1,4]. *M*_ox_ and *M*_red_ are the oxidized and reduced forms of the mediator. *E*_ox_ and *E*_red_ are the oxidized and reduced enzyme. *S* and *P* are the substrate and product of the enzymatic reaction. *D*_E_ and *D*_M_ are the diffusion coefficients of enzyme and mediator respectively; the coefficients of the oxidized and reduced forms are assumed to be equal. Oxidized mediator Os(III) is only produced at the electrode surface by re-oxidation of Os(II).

**Scheme II:**
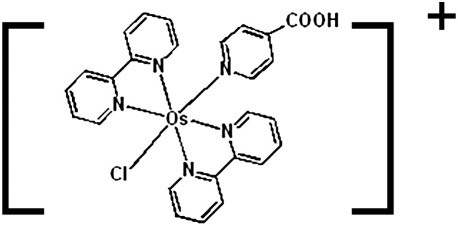
The structure of [Os(bpy)_2_ClPyCOOH]^+^.

**Table 1:** Comparison of kinetic constants obtained by approximate analytical solutions and simulation

	Eq. (6)	Eq. (5)	Simulation
*K*_MS_ (mM)	9.3 ± 2.5	–	10.8 ± 1.4
*k*_cat_ (s^− 1^)	233 ± 22	–	254 ± 20
*K* × 10^5^ (M^− 1^ s^− 1^)	–	2.1 ± 0.2	2.2 ± 0.2
*k*_cat_/*K*_MS_ (× 10^4^ M^− 1^ s^− 1^)	2 ± 1	–	2.3 ± 0.4

**Table 2:** Comparison of kinetic data for homogeneous catalysis of glucose oxidase with different soluble redox mediators at pH = 7 (unless otherwise specified)

Mediator	*k*_cat_/s^− 1^	*K*_MS_/mM	*k*/M^− 1^ s^− 1^	*k*_cat_/*K*_MS_ M^− 1^ s^− 1^
O_2_[29, 36]	900	68	1.6 × 10^6^	1.32 × 10^4^
O_2_[30] (pH not specified)	NR	around 20	NR	NR
O_2_[35] (pH = 5.6)	NR	33	NR	NR
Ferrocenemethanol [39]	780	65	6.0 × 10^6^	1.20 × 10^4^
Ferrocenemethanol [40]	400	42	2.0 × 10^6^	0.95 × 10^4^
Ferrocenemethanol [32]	408	36	NR	1.13 × 10^4^
Ferrocenemonocarboxylic [39]	NR	NR	1.5 × 10^5^	NR
Ferrocenemonocarboxylic [1]	497	29	2.2 × 10^5^	1.70 × 10^4^
Ferrocenemonocarboxylic [8]	NR	NR	6.0 × 10^4^	NR
Ferrocenemonocarboxylic [6]	NR	NR	2.0 × 10^5^	NR
Ferrocenedimethanol [32]	340	30	NR	1.13 × 10^4^
(ferrocenemethyl)Dimethylammonium [39]	NR	NR	1.0 × 10^7^	NR
(ferrocenemethyl)Trimethylammonium [32]	233	27	NR	8.6 × 10^3^
Ferrocenemonosulfonate [8]	95	88	9.5 × 10^4^	1.1 × 10^3^
