# Supplementary material for: Extracting kinetic parameters for homogeneous [Os(bpy)2ClPyCOOH]+ mediated enzyme reactions from cyclic voltammetry and simulations
Source: Bioelectrochemistry. 2008 Nov;74(1):201–9. doi: 10.1016/j.bioelechem.2008.08.001 (PMC2742741; doi:10.1016/j.bioelechem.2008.08.001)
Supplement: Supplementary file 1 [file mmc1.doc]

**Extracting kinetic parameters for homogeneous [Os(bpy)_2_ClPyCOOH]^+^ mediated enzyme reactions from cyclic voltammetry and simulations**

|

V. Flexer^(1)^, M.V. Ielmini^(1)^, E.J. Calvo^(1)^ and P.N. Bartlett^(2)^

(1) INQUIMAE, Departamento de Quimica Inorganica, Analitica y Quimica Fisica, Facultad de Ciencias Exactas y Naturales, Universidad de Buenos Aires, Buenos Aires. Argentina

(2) School of Chemistry, University of Southampton, Southampton, Hants SO17 1BJ, UK

## Figure S-1

**Figure S-1** UV-Vis spectra of the measurement solution, showing that the [Os(bpy)_2_Cl(PyCOOH)] concentration is unaltered during the course of the experiment. Full line, the solution contains only buffer, GOx and [Os(bpy)_2_Cl(PyCOOH)]^+^. Dotted line, the enzyme has been in contact with increasing concentrations of D-glucose for 40 minutes. Note that both spectra are almost indistinguishable.

**Figure S-2**

# Figure S-2 Plot of the current as a function of glucose concentration in case VI. *m*_Σ_ =1.02mM, *e*_Σ_=1.6μM in NaH_2_PO_4_/Na_2_HPO_4_ 0.5M + 0.1M NaCl buffer solution. The line shows the best fit to eq. (5) of the experimental data.

# Figure S-3

**Figure S-3** Plot of the peak current for [Os(bpy)_2_ClPyCOOH]^+^ as a function of the square root of the scan rate both in the presence of 100mM D-glucose (open circles) and without (black dots). The plot shows that the diffusion coefficient for the Os complex does not change in the presence of glucose in buffer solution.

**Figure S-4**

**Figure S-4** Plots of the current as a function of time and as a function of potential in case VI for GOx with a different redox mediator: ferrocene methanol. *m*_Σ_ = 4.7mM, *e*_Σ_=1. 5μM and *s*_∞_ = 70mM in NaH_2_PO_4_/Na_2_HPO_4_ 0.1M + 0.1M NaCl buffer solution. *a*) Chronoamperometry experiment; b) two successive voltammograms, second voltammogram 1 hour after the first one.

**Figure S-5**

**Figure S-5** Plot of the current as a function of glucose concentration in case VI with an enzyme from a different origin, Medisense®, UK. *m*_Σ_=1.02mM, *e*_Σ_ = 1.8μM in NaH_2_PO_4_/Na_2_HPO_4_ 0.5M + 0.1M NaCl buffer solution. The line shows the best fit to eq. (5) of the experimental data.
